# The Role of Transcriptional Regulation in Hybrid Vigor

**DOI:** 10.3389/fpls.2020.00410

**Published:** 2020-04-15

**Authors:** Ramon Botet, Joost J. B. Keurentjes

**Affiliations:** ^1^Laboratory of Genetics, Wageningen University & Research, Wageningen, Netherlands

**Keywords:** heterosis, hybrid vigor, gene expression, genetic regulation, speciation, breeding, natural variation, *Arabidopsis thaliana*

## Abstract

The genetic basis of hybrid vigor in plants remains largely unsolved but strong evidence suggests that variation in transcriptional regulation can explain many aspects of this phenomenon. Natural variation in transcriptional regulation is highly abundant in virtually all species and thus a potential source of heterotic variability. Allele Specific Expression (ASE), which is tightly linked to parent of origin effects and modulated by complex interactions *in cis* and *in trans*, is generally considered to play a key role in explaining the differences between hybrids and parental lines. Here we discuss the recent developments in elucidating the role of transcriptional variation in a number of aspects of hybrid vigor, thereby bridging old paradigms and hypotheses with contemporary research in various species.

## Introduction

Hybrid vigor is the phenomenon of an improved performance of the progeny of a cross between two different parental genotypes over these genotypes. Many of the improved characteristics are directly related to plant physiology and adaptation or agriculturally important developmental and reproductive traits, such as growth vigor or seed yield. However, heterozygous genotypes also appear to perform better and reproduce more successfully in variable environments as demonstrated by higher yield stability of hybrids than inbred lines in cereals (Betran et al., 2003; Mühleisen et al., 2014). The mechanism of hybrid vigor thus has a major impact, both in evolution and agriculture.

From an evolutionary perspective, hybrid vigor may lead to the formation of new species. Similar to intraspecific crosses, increased vigor in performance and reproductive traits is frequently observed after hybridization events between different allogamous species. Because phenotypic changes are caused by increased genetic heterogeneity in both cases, it is hypothesized that regulatory mechanisms may be similar. Illustratively, some recently reported invasive plant species derived from combinations of heterozygous polyploids, and domesticated species such as wheat emerged from interspecific crosses and fixation by ploidy changes (Chen, 2010; Storme and Mason, 2014).

Since its discovery in maize (*Zea mays*), hybrid vigor has been intensively exploited in a wide range of highly valuable crops. First reported by Darwin (1876), hybrid vigor has since been an object of research and debate in the scientific community and many studies still shed new light on the topic on a regular basis.

Many hypotheses on the genetic regulation of hybrid vigor have been formulated over the last century, which can be summarized by a combination of additive and dominance effects and additional epistatic interactions. From an agricultural perspective hybrid vigor is often referred to as heterosis, although, strictly speaking, heterosis is the component of hybrid vigor resulting exclusively from the effect of heterozygous loci, such as allelic complementation and over-dominance. Additive effects of homozygous loci, and epistatic interactions between homozygous and/or heterozygous loci are considered to constitute major other components of hybrid vigor.

Despite a single exception reported in tomato, in which a single functional Mendelian locus explained hybrid vigor in reproductive traits (Semel et al., 2006), in most species hybrid vigor appears to have a polygenic origin represented by large, highly interconnected, gene regulatory networks (GRN) (Brieger, 1950; Lober, 1968; Stuber et al., 1992), and disentangling its molecular basis is, therefore, still challenging. In rice, for instance, different loci explained proportions of yield heterosis in hybrids derived from diverse founders (Huang et al., 2016). Next to the general believe of functional complementation of deleterious alleles, the relatively novel insight of transcriptional regulation of hybrid vigor is gaining support. Allele specific expression (ASE) analysis is a very accurate way of determining how genetic effectors are expressed in a given genetic background. *Trans-*regulatory variation controlling the intensity of expression of a given gene, and specific modulation of the expression of multiple alleles *in cis*, are common factors thought to explain most of the transcriptional variation. Technological advances involving RNA-seq have overtaken traditional technologies based on microarray hybridization and cDNA library (e.g., Expressed Sequence Tag) sequencing and a considerable number of highly valuable transcriptomics studies have recently been conducted, which we believe need to be reviewed. In the following sections we aim to explain and decompose how transcriptional regulation affects hybrid vigor, highlighting the most influential studies.

## Natural Variation in Gene Expression

**FIGURE 1:**
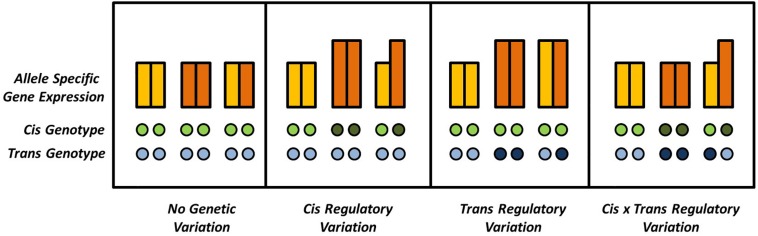
Possible combinations of *cis* and *trans* effectors affecting a given locus. Gene expression is quantified by the bars, where different colors indicate different alleles of a given diploid locus. Differences in ASE can be absent or explained by (interactions of) *cis* and *trans* effectors. Absence and presence of genetic variation in *cis* or in *trans* is indicated by open and solid circles, respectively.

Variation in gene expression is one of the most intriguing phenomena in modern biology and can be observed across tissues and developmental states or be modulated by the environment (Emerson et al., 2010; Metzger et al., 2016; Soyk et al., 2017). In addition, natural variation for gene expression can be observed in different genotypic backgrounds. In hybrids, genes can occur in either homozygous or heterozygous pairs. Homozygous genes might be differentially expressed in different genetic backgrounds, e.g., the hybrid and its parents, indicating *trans-*regulatory variation contributing to additive effects on the phenotype. The different alleles of heterozygous genes, on the other hand, can in addition be differentially expressed within the same genetic background, indicating *cis-*regulatory, or allele specific, expression variation. Mutations acting *in trans*, although exhibiting smaller effects, are usually much more frequent than the more effective *in cis* mutations, simply because of the polygenic inheritance patterns of expression regulation (Figure 1), as was recently demonstrated in yeast (Metzger et al., 2016).

*Trans-*regulatory variation in gene expression is indirectly caused by allelic variation in modifiers and other expression regulators, such as transcription factors. This variation can be functional, e.g., through coding sequence variation, or transcriptional, affecting the abundance of the regulator. By definition, *trans-*regulatory genes are located at physically different positions than their target genes, which can be very distant or even located on different chromosomes. *Trans-*acting variation is widely distributed among regulatory networks and the expression potential of genes very often has a multigenic inheritance similar to complex phenotypes. This is due to the pleiotropic nature of many regulators, affecting the expression of many genes, and the presence of numerous *cis-*elements in promoter sites of genes, allowing them to be targeted by multiple regulators. It is likely that the heterogeneous background of hybrids, compared to inbred lines, provides a much richer palette for *trans-*regulatory variation, widening the opportunity for fine-tuning the transcriptional program.

*Cis-*regulatory variation is defined by allelic variation of the expressed gene itself. Variation between genotypes at the genetic level can be introduced by coding sequence polymorphisms that lead to structural changes in the resulting mRNA and translated protein. These changes might modify mRNA and protein stability and protein activity, which may result in phenotypic differences or feedback to transcriptional control. In addition, polymorphisms in *cis-*regulatory promoter elements may directly affect the expression level of a gene, e.g., by impairing or introducing transcription factor binding sites. Similar to the increased *trans-*regulatory variation in hybrids, *cis-*regulatory variation in heterozygous genes may contribute to the optimization of transcriptional programs. Illustratively, in yeast *cis* mutations in one allele disclose an increased effect on the average bi-allelic gene expression, indicating a greater chance to influence the phenotype and respond to natural selection (Emerson et al., 2010; Metzger et al., 2016).

Finally, cis × *trans* interactions occur when genotypic variation at an unlinked locus (*in trans*) is required to read out allele-specific effects *in cis*, further increasing the regulatory capacity of heterogenic individuals. Excellent examples have recently been reported in tomato hybrids, where yield was modulated by fine-tuning the expression of a MADS-box transcription factor and its *trans* effects on natural and engineered alleles (Soyk et al., 2017).

The genetic control of transcription and gene interaction can be effectively studied by expression quantitative trait locus (eQTL) analysis in segregating populations and represented in GRN, which are linked with system-wide phenotypic effects in Arabidopsis (Fu et al., 2009). GRNs provide a wider perspective on the transcriptional program and can be instrumental in the assignment of candidate genes involved in the regulation of complex phenotypic traits (West et al., 2007; Fu et al., 2013; Zhai et al., 2013). Moreover, eQTL studies allow the reconstruction of complex trait regulation by integrating the genetic control of expression variation with the segregation of phenotypic variation. For instance, eQTL hotspots indicate the presence of master *trans* regulators, which are believed to have a strong influence on the phenome (Holloway et al., 2011; Wang et al., 2014). Most recent eQTL studies have moved from microarray analysis of segregating biparental populations to the more comprehensive coupling of RNAseq technologies with genome wide association (GWA) mapping panels (Cookson et al., 2009; Zhang et al., 2011; Kawakatsu et al., 2016). Next to the wider range of phenotypic diversity analyzed, encompassed by the larger number of genotypes interrogated, the much higher resolution of GWA mapping allows to distinguish true *cis-*regulation from *trans-*effects on linked loci, further contributing to the accurate assignment of candidate genes. However, higher genetic diversity doesn’t always indicate wider phenotypic variation, especially if the population was subject to constraining selection.

Although most eQTL studies are carried out in homozygous inbred populations, their discoveries may be highly relevant for the dissection of hybrid vigor traits as the regulation of gene expression can be translated into additive-like genetic mechanisms. In that respect, an optimal combination of specific homozygous alleles may thus lead to a similar output than that obtained from an heterozygous genotype. Interestingly, recurrent selection breeding in *Arabidopsis thaliana* leads to homozygous inbred lines that perform similar to vigorous hybrids (Wang et al., 2015). While it could erroneously be assumed that dominance effects may, therefore, not be important in hybrid vigor in Arabidopsis, the hybrid mimicry could just be the product of selection for optimal combinations of additive effects. This indicates that segregation of sufficient genetic variation and the appropriate combination of *cis-* and *trans-*effectors in homozygotes are as important as variation resulting from ASE achieved in heterozygous loci.

## Allele Specific Expression

Allele Specific Expression is defined as the differential expression of the two alleles of a gene in a hybrid in a particular developmental stage, tissue or organelle or in response to an environmental stimulus and may be one of the most important effector mechanisms accounting for hybrid vigor. ASE allows for a wider phenotypic variation due to the increased plasticity of gene expression governed by diverse alleles. In addition, functional complementation in a hybrid can be the result of ASE, when preferentially silenced deleterious alleles are complemented by an overexpressed beneficial allele. In contrast to the expression regulation of homozygous alleles, which occurs solely through *trans-*regulators, ASE concerns heterozygous alleles, whose differential expression is largely controlled by *cis-*acting genetic variation, although *trans-*acting variation interacting with the allelic state does occur as well.

Genetic mechanisms, such as dominance and additivity, causing hybrid vigor through heterozygous loci can biologically be explained by the modulation of ASE. In maize hybrids, for instance, gene expression complementation of genomic absence in one of the parents occurred, consistent with the dominance model for hybrid vigor (Paschold et al., 2012). The level of expression of the different alleles in a hybrid ranges from overexpression to complete silencing and determines its mechanistic classification. Genes of which both alleles are higher expressed in the hybrid than in both the founding parents represent one extreme of the spectrum. The expression of such genes is a classic example of overdominance, which might be caused by the change in genetic background. Likewise, the partial or complete silencing of both alleles in the hybrid, in contrast to normal expression levels in its parents is referred to as underdominance. Underdominance represents the other end of the spectrum and is related to negative heterosis. Both over- and under-dominance of gene expression in hybrids can be explained by additive or epistatic *trans-*regulation, which is absent or incomplete in the parental lines. Intermediate expression levels of genes occur when both alleles are expressed at a similar rate in the hybrid but differentially in the parents. In this case the different alleles act completely additive, resulting in codominance with potential beneficiary effects in hybrids. Finally, preferential allelic expression, with the extreme case of monoallelic expression, results in an intermediate but genotypically skewed gene expression, usually classified as partial dominance. The differential expression of alleles in a hybrid may resemble the difference in expression levels of those alleles in the homozygous parents, in which case ASE is most likely caused by *cis-*regulation and hybrid vigor might result from functional complementation. However, in the case of preferential silencing in the hybrid but comparable expression levels in homozygous lines, cis × *trans* interactions might be involved as well.

It has been speculated that, because of the difficulty in mapping due to polygenic heterozygosity, hybrid vigor might not be explained by specific loci but rather should be considered as a whole-genome phenomena. Early studies in maize, Arabidopsis and rice (*Oryza sativa*) have clearly demonstrated the relative contribution of genome-wide ASE to hybrid vigor (Li et al., 2016). These pioneering studies determined ASE by assessing the transcriptional allelic ratio in F_1_ hybrids and comparing this to the parental expression levels, an approach which has also been applied for interspecific crosses in Drosophila (Wittkopp et al., 2004). Because each allele is analyzed in a unique background (homozygous parents) as well as in an identical background (F_1_ hybrid), such a strategy allows to discriminate between true *cis-* and *trans-*acting variation. Initial genome-wide studies revealed that more than 40% of the differentially expressed genes (DEGs) in parental lines displayed ASE in hybrids, indicating the impact of *cis-*regulatory variation at heterozygous loci (Zhang and Borevitz, 2009). In addition, a significant enrichment of DEGs can be observed in QTL regions explaining variation in hybrid vigor (Wei et al., 2009). Moreover, the strong correlation between differential gene expression and hybrid performance enables the prediction of hybrid vigor by examining transcriptional activity at the parental level (Thiemann et al., 2010). Although these studies are based on very few genotypes, and extrapolating conclusions to other genotypes and plant species might not always be appropriate, the identified associations emphasize the relevance of transcriptional regulation in the control of hybrid vigor.

More recent studies, now based on RNA-seq, attempted to quantify the type of ASE that is associated with hybrid vigor. Interestingly, the proportional distribution of ASE types in two rice F_1_ hybrids, with contrasting vigor levels, was practically identical. However, an overlap in ASE genes was almost absent (Song et al., 2013). This indicates that, rather than the type of expression of heterozygous loci, the sort of genes that are differentially expressed explain hybrid vigor better. Nonetheless, although background interactions via *trans* effects were not accounted for, biallelic expression was observed in almost three quarters of the genes. This is in agreement with one of the first ASE studies in maize, which examined differences between old and new hybrid varieties, adapted to early and more recent crop management practices, respectively (Guo et al., 2004). Although a small set of genes in a limited number of genotypes was interrogated, a remarkable shift from monoallelic expression in the older varieties toward a biallellic type of ASE in more contemporary varieties was observed. Biallelic ASE offers the advantage of a larger arsenal of opportunities for adaptation to changing and new environments. Even though shifts in monoallelic expression between alleles of a given loci might provide a similar advantage, observations of this kind of regulation have not been reported so far.

In agriculture, adaptation to new environments includes the expansion of a given crop beyond its normal cultivation areas and, more interestingly, a better adaptation to fluctuations in the environment, which may occur during the growing season (Betran et al., 2003). In light of their increased vigor it has been hypothesized that most gains from hybrids are due to genetic improvement of plant performance in stress conditions (Duvick, 2001; Betran et al., 2003). However, studies in different species remain inconclusive of increased stability of hybrids but suggest that robustness may be related to the species reproductive system (Stelling et al., 1994; Bruns and Peterson, 1998; Mühleisen et al., 2014; Liu et al., 2017; Zhang et al., 2017). Genotype by Environment (GxE) interactions are commonly addressed in QTL studies and determine how genetic polymorphisms can explain variation in a given trait in specific environments. Similarly, ASE can be analyzed in multiple environments by quantifying GxE interactions at the transcriptional level. Such studies suggest a significant effect of GxE interactions on the regulation of the expression level, both *in cis* and *in trans*, although *trans* GxE effects are much more robust to environmental variation (Cubillos et al., 2014). As such, environmental modulation of transcriptional networks may lead to improved responses to these stimuli.

Relating ASE to hybrid vigor has greatly increased our understanding of the effects of differential gene expression at the phenotypic level of hybrids. However, the complexity of this relationship increases when the historical progress made in hybrid breeding is taken into account and potential causal effectors such as environment interactions are incorporated.

## Parent of Origin Effects

Parent of origin effects are referred to as transgenerational effects that are imposed by the paternal or maternal genetic makeup, independently from the progenies own genotype. As such, the genotype of the parents of a hybrid can have substantial impact on the hybrid’s transcriptional program and hence contribute to hybrid vigor. Estimating parent of origin effects can be extremely challenging since these can not only be affected by the parental genotype but also by their physiological state, reflecting the environmental growing conditions. Two main sources for transgenerational effects are generally distinguished: maternal effects and genomic imprinting.

Maternal effects are based on physiological properties expressed in the mother plant, which are passed on to their progeny, independent of that progeny’s genotype. That said, the inheritance of chloroplasts and mitochondria are usually also classified as maternal effects though having also a strong genetic component. Maternal traits in plants are predominantly related to seed formation due to the maternal genotype of the endosperm, which can strongly affect early stages of development. In Sorghum, the analysis of parent-of-origin expression patterns in the endosperm of hybrids revealed that among genes with ASE the vast majority overexpressed the maternal allele (Zhang. X. et al., 2016). Although it is tempting to include the maternal genotype as effector in hybrid vigor based on its contribution at the chloroplast and mitochondrial level, early stage effects derived from seed related variation could be key for the establishment of young seedlings and explain hybrid vigor at later stages. Inheritance of such traits occurs via the gametophyte through maternal tissues such as the embryo sac, which will develop into a 3n endosperm or via the sporophyte, when the haploid maternal and paternal gametes will fuse into a 2n embryo. Dosage sensitive loci or mutations are likely candidates to cause expression variation in these reproductive tissues. In addition, cytoplasmic effects have been well documented, which can be an added source of phenotypic variation (Kihara, 1982; Edwards et al., 1996; Flood et al., 2020). In most plant species cytoplasmic organelles are strictly inherited from the female, although some species, e.g., cucumber, show a paternal mitochondrial inheritance (Havey, 1997). The direction in which a cross is made can, therefore, have strong consequences for the transcriptional program of offspring, as can be observed in reciprocal hybrids. Moreover, changes in cyto-nuclear interactions can greatly affect growth and development in plants (Joseph et al., 2015; Flood et al., 2020).

While maternal effects, by definition, are solely caused by female inheritance, genomic imprinting can also have a male origin. Genomic imprinting refers to epigenetic marks that can be transferred from the parental lines onto the progeny’s genome, adding an extra level of complexity to transcriptional regulation. Imprinting effects were first reported in maize, in which the distribution of anthocyanin in the aleurone layer of the endosperm depends on the genotype of the parents in addition to the direction of crossing (Kermicle, 1970). Even though genomic imprinting has been shown to be causal of very specific phenotypic differences, a much more comprehensive view of its effects can be obtained by studying DEGs as a phenotype. Differences in the expression of parent-of-origin alleles are typically measured in reciprocal hybrids at heterozygous loci and monoallelic expressed genes are categorized as Maternally Expressed Genes (MEGs) or Paternally Expressed Genes (PEGs) depending on which allele is expressed.

In addition to *cis-*elementary variation, monoallelic expressed genes are assumed to be influenced by differences in methylation patterns of the alternative alleles, suggesting distinct activation pathways (Hatorangan et al., 2016). In contrast to this, recent evidence suggests that DNA-methylation variation might also result from gene expression differences, particularly transient DNA methylation changes at adjacent repetitive elements of stress induced genes (Secco et al., 2015). Interestingly, MEGs occur more frequently than PEGs (Springer and Stupar, 2007). This apparent discrepancy might be explained by a sex-related difference in DNA demethylation during zygote formation causing a higher activation of maternal alleles, while paternal alleles would remain silenced for a longer time (Waters et al., 2011). Even though some loci might escape entirely from maternal silencing, such as during seed development (Vielle-Calzada et al., 2001), partial imprinting might be more common than complete imprinting (Gregg et al., 2010), possibly due to a rather attenuated than completely silenced paternal gene expression (Weijers et al., 2001).

Despite the small number of genes affected by genomic imprinting and preferential gene expression being limited to early developmental stages (Guo et al., 2004; Springer and Stupar, 2007; Cubillos et al., 2014), parent of origin effects in hybrids may have prolonged effects in later tissues. Preferentially expressed genes are often part of large gene regulatory networks and polygenic regulation is common for many complex traits. Therefore, strategic positioning of preferentially expressed genes as central hubs in highly interconnected GRNs may have strong radiating effects on a plethora of traits. Moreover, imprinted genes may be differentially regulated at subsequent stages of development, allowing adequate responses to external stress, which would be highly relevant regarding the adaptation to multiple environments (Sanchez and Paszkowski, 2014; Groszmann et al., 2015). Curiously, the degree of conservation of imprinted regions between species is relatively low (Waters et al., 2011; Zhang. M. et al., 2016) and preferentially expressed genes are more prone to evolve due to a higher proximity to transposable elements (Waters et al., 2011; Wolff et al., 2011; Hatorangan et al., 2016). The increased allelic variation of preferentially expressed genes might, therefore, have a larger impact on hybrid vigor than would be expected from their incidence (Eichten et al., 2013; Al Adhami et al., 2015). Similarly, increased diversity might suggest a significant evolutionary advantage in speciation events, although it has been hypothesized that imprinting could also be a barrier for interspecific hybrid formation due to genetic incompatibilities (Wittkopp et al., 2004; Zhang and Borevitz, 2009).

## Epigenetic Regulation of Hybrid Vigor

Epigenetics is in addition to DNA-sequence variation a higher order mechanism for the regulation of gene expression (Jenuwein and Allis, 2001; Kouzarides, 2007). Epigenomes are specified by DNA methylation and histone modification differences, which selectively expose or hide a targeted sequence from the transcriptional machinery. This can activate or repress the expression of a specific gene and modulate ASE at heterozygous loci. Dissimilarities in methylation patterns of loci between lines are referred to as epi-alleles or differentially methylated regions (DMRs), which can be stably inherited over many generations (Greaves et al., 2012; Schmitz et al., 2013; Kooke et al., 2015). In hybrids, the inherited epigenetic marks can either exhibit an additive or a non-additive effect. Non-additive effects arise when one of the parental’s methylation profile is copied to the complementary DNA strain, resulting in homozygous methylation patterns. In contrast, sustained epigenetic differences between homologous chromosomes lead to a unique heterozygous epigenome and additive allele specific differences (Yang et al., 2016). Although epigenetic variation is often accompanied by genetic DNA sequence variation (Secco et al., 2015), there are strong indications that epigenetic mechanisms can substantially contribute to explaining hybrid vigor (Kawanabe et al., 2016). For example, hybrids derived from inbred lines of high genetic resemblance but divergent for their epigenomes are still capable of producing high heterotic levels (Meyer et al., 2004; Kawanabe et al., 2016). Furthermore, epigenetic modulation at the transcriptional level may provide an appropriate response to environmental factors, or developmental processes at vegetative and reproductive stages to optimize a genotype’s performance in changing conditions (Kawakatsu et al., 2016). An extensive list of studies have uncovered several mechanisms for this type of regulation [see (Groszmann et al., 2013; Greaves et al., 2015) for excellent reviews on the topic]. In summary, epigenetic variation contributes to heterosis by exploiting allelic diversity through regulation of ASE variation, possibly via parent of origin effects or in response to environmental cues.

## Fixation of Hybrid Vigor: Polyploidy and Speciation

Hybrids exhibit clear advantages in particular environments and selective forces might drive evolution to preserve these advantages for the immediate offspring. One way through which hybrid vigor is naturally fixed occurs in admixed populations, where optimal allele frequencies of heterotic loci are maintained by increased natural intercrossing of heterogeneous individuals with higher fitness (Oakley et al., 2015).

Another mechanism of fixing hybrid vigor is through polyploidization, possibly followed by a diploidization event (Chen, 2010). Changes in genome dosage involve an increase in chromosome numbers, usually caused by an abnormal meiosis leading to unreduced gametes. Unreduced gametes are characterized by having an aberrant number of chromosomes, that after fertilization will produce a zygote with a different genome dosage. If the change of genome dosage involves the whole chromosome set, the zygote will display a change of ploidy, whereas the offspring will be defined as an aneuploid if only one or a few chromosomes are duplicated. Polyploids tend to show increased performance and larger sizes, which ultimately may translate into a reproductive increase (Gu et al., 2003; Comai, 2005; Orr-Weaver, 2015). In addition, changes in ploidy can fix heterotic loci when a polyploidization event occurs in a hybrid, especially when the homeologous chromosomes of the resulting polyploid are inherited disomically, i.e., without recombination. This might explain why most plant species have gone through polyploidization events, which ultimately lead to higher fitness and adaptation (Soltis et al., 2009; Vanneste et al., 2014). Moreover, invasive species are more likely to be derived from polyploidization events, which would suggest a better adaptation to different environments as well (Pandit et al., 2011; te Beest et al., 2012).

In contrast to many polyploids, aneuploids are genetically unstable, producing gametes with variable chromosome numbers. These aberrant gametes are incompatible with normal gametes from the same species but might allow the mating with gametes of similar chromosome number from other related species (Vallejo-Marin et al., 2015). Even though hybrid vigor itself might not affect evolution directly, many indications suggest that it could support maintaining and increasing fitness of intermediate genotypes going through speciation events (Storme and Mason, 2014). Interspecific crosses are a possible mechanism by which hybrid vigor can be conserved but are also the source of incipient speciation. Because recombination between the different homeologs does usually not occur and crossing with its ancestors is impaired, heterozygous loci are fixed in the polyploid’s progeny. Illustratively, related polyploid forages provide extremely high yields, which may partly be explained by such fixed heterotic loci (East, 1936; Brummer et al., 1999). Thus, polyploidization is a means of fixing hybrid vigor in intercrosses and may serve as an initiation for speciation.

## Discussion

Ongoing research keeps enforcing the role of transcriptional variation in explaining hybrid vigor, with studies ranging from well-established model species to high value commercial crops. Transcriptional control is a key regulation mechanism explaining hybrid vigor. This can be envisaged by a complex and genotype-unique network of small effects and interactions derived from natural variation in gene sequences and their ASE in addition to the derived complex interactions by *cis* and *trans* acting elements. All those features have recently been shown to be a powerful tool in fine-tuning the plant response to environmental changes and ultimately leading to a better performance.

A major pillar of species evolution, and plant breeding in the slipstream thereof, is the generation of and selection for genetic variation. From an evolutionary perspective, natural variation allows the development of traits that might provide an adaptive advantage in changing environments, while breeding focuses on the yield and quality of harvestable products. Although much of the observed phenotypic variation may result from allelic differences in the function of genes, due to genetic variation in coding sequences, a substantial proportion can be explained by differences in gene expression. Gene expression variation can be caused by sequence variation in regulatory regions of the DEG (*in Cis*) or by functional variation in a regulator (*in Trans*). In hybrids and outcrossing species many genes occur in a heterozygous state, which offers an increase in allelic variation. Indeed, in hybrids many genes are differentially expressed compared to their parents, explaining an important part of hybrid vigor. Genes might be differently expressed in response to a change in genetic background irrespective of the allelic state of the gene. However, in many cases genes display ASE, in which one allele is preferentially expressed over the other. This occurs when gain or loss in *cis-*regulatory elements results in changes in transcriptional activity. If the different alleles also harbor variation in function this may provide an advantage in certain conditions. A possible mechanism underlying ASE may be parent-of-origin effects, in which there is a preference for the expression of one of the parental alleles. This can be a consequence of physiological effects or epigenetic imprinting of parental alleles. Parent-of-origin effects are most profound in seeds developing on the mother plant, which might explain why more DEGs are maternally expressed. One of the longstanding paradigms is that heritable genetic variation must consist of DNA sequence variation. However, compelling evidence increasingly indicates that epigenetic variation can be transferred transgenerational and as such may be a source for selectable phenotypic variation. Undeniably, epigenetic regulation may be a cause of differential gene expression and, therefore, might be partially responsible for any observed hybrid vigor. Given the clear advantage of hybrids in natural settings it can be envisaged that selection favors the maintenance of hybrid vigor by increasing the likelihood that heterozygosity prevails in individuals of admixed populations or by fixing the occurrence of multiple alleles in a single individual by polyploidization. Indeed, polyploids often represent the first stage of incipient speciation, which might reflect the beneficial properties of increased genetic variation. Similar mechanisms might account for the relatively large success of hybrids in breeding strategies. Hybrid vigor can be decomposed into several components: additive effects, heterosis effects, and their resulting interactions. However, over the last decade maize breeding companies have noticed slightly diminishing heterosis levels achieved year after year. This may be caused by a better performance of the parental lines, upon which heterosis is estimated. Notably though, when hybrids are compared across years genetic improvement can still be observed. A few observations may, therefore, condition future hybrid breeding. Genetic gain has occurred faster in parental lines than in hybrids, indicating additive components as major effectors in current models of hybrid vigor. Traditional hybrid breeding programs where heterozygosity is maximized, limits the opportunity to target those regions. Recurrent selection models aiming at stacking additive components might establish fixation of pre-hybrid vigor in the parents. Furthermore, the contribution of epistasis to hybrid vigor is largely unknown but assumingly significant. This is exemplified by a strong background effect when heterosis QTLs are introgressed in different genotypes. Investigating the transcriptional networks underlying hybrid vigor might thus reveal the mechanistic basis and genetic architecture of hybrid vigor in natural and artificial settings. Whilst the union of transcriptomics and hybrid vigor research has already obtained numerous novel insights, new questions keep arising, which make future research an exciting field to dive in.

## Author Contributions

RB and JK conceptualized and wrote the article with equal input.

## Conflict of Interest

The authors declare that the research was conducted in the absence of any commercial or financial relationships that could be construed as a potential conflict of interest.
